# Enzymatic potential and biosurfactant production by endophytic fungi from mangrove forest in Southeastern Brazil

**DOI:** 10.1186/s13568-019-0850-1

**Published:** 2019-08-19

**Authors:** Vivian Martinho, Lidiane Maria dos Santos Lima, Caroline Almeida Barros, Vitor Baptista Ferrari, Michel Rodrigo Zambrano Passarini, Leonardo André Santos, Fernanda Luisa de Souza Sebastianes, Paulo Teixeira Lacava, Suzan Pantaroto de Vasconcellos

**Affiliations:** 1 Department of Pharmaceutical Sciences, Federal University of São Paulo (UNIFESP), R. São Nicolau, 210, Diadema, SP Zip Code 09913-030 Brazil; 2 Latin American Institute of Life Sciences and Nature, Federal University of Latin American Integration, Av. Tarquínio Joslin dos Santos, 1000, Foz do Iguaçu, PR Zip Code 85870-901 Brazil; 3 Laboratory of Microbiology and Biomolecules - LaMiB, Department of Morphology and Pathology, Center for Biological and Health Sciences, Federal University of São Carlos, Via Washington Luís km 235, PO BOX 676, São Carlos, SP 13565‑905 Brazil

**Keywords:** Bioethanol, Biosurfactant, Cellulases, Ligninases, Xylanases

## Abstract

Microbial activity is the main route for cycling mangrove nutrients. In general, microorganisms have abilities to degrade lignocellulosic compounds. Among the biotechnological potential of the microbiota from mangroves, it is noteworthy about endophytic fungi, which can be considered as effective sources of different bioactive compounds. In this sense, thirty (30) endophytic fungi were isolated from mangrove forest sampling Cananeia, SP, Brazil. These microorganisms were analyzed about their enzymatic activities including: lignin peroxidase EC 1.11.1.14, manganese peroxidase EC 1.11.1.13 and laccase EC 1.10.3.2, as well endo-cellulase EC 3.2.1.4 and endo-xylanase EC 3.2.1.8. Besides that, production of bioactive secondary metabolites like biosurfactant and/or bioemulsifier was also investigated. As results, nineteen (19) isolates were selected about their ligninolytic abilities, nine (9) of them about cellulase activity and thirteen (13) showed xylanase abilities. The fungal isolate named as 3(3), characterized as *Fusarium sambucinum*, showed a prominent lignin peroxidase (42.4 U L^−1^) and manganese peroxidase (23.6 U L^−1^) activities. The isolate 63.1, also related to *Fusarium* sp. genera, was selected about its laccase activity (41.5 U L^−1^). From all the investigated fungi, the isolate 47(4) *Trichoderma camerunense* was selected about its cellulolytic and xylanolytic activities, showing 45.23 and 26.09 U mL^−1^, respectively. The same fungi also showed biosurfactant ability demonstrated by superficial tension decreasing to 38 mN/m. In addition, fifteen (15) fungi exhibited bioemulsifier activity, with E_24_ values up to 62.8%.

## Introduction

Mangroves are coastal ecosystems with high production of organic matter to adjacent coastal waters (Badola et al. 2008). It is estimated that they cover around 18.1 million ha worldwide (Andreote et al. 2012). Microbial activity is the main route of nutrient cycling in mangroves (Kristensen et al. 2008; Sousa et al. 2006). Among different types of microorganisms at mangroves is worth mention about the endophytic fungi, whose can establish mutualistic associations with plants (Schwarz et al. 2004).

These microorganisms can exhibit biochemical versatility and biological diversity, which have revealed dense variety of genes with important biotechnological applications (Sebastianes et al. 2013), including the production of ligninases, cellulases and xylanases (Zheng et al. 2016; Bezerra et al. 2012; Rajulu et al. 2011).

Conventional ethanol production or first-generation ethanol is produced by fermentation of sugarcane juice by *Saccharomyces cerevisiae* (Peixoto et al. 2012). Although the production of second-generation ethanol, or lignocellulosic bioethanol from biomass, are processes that need efficient enzyme-producing microorganisms to perform the extraction of all polysaccharides. For this, it is required the use of different enzymes, including oxidases and hydrolases (Manavalan et al. 2015; Aguiar and Ferraz 2011).

Lignin peroxidases (EC 1.11.1.14) are able to oxidize benzyl alcohols, breakdown aromatic chains, perform intramolecular rearrangements, and break rings into non-phenolic compounds related to lignin (Rabonato 2013). They can be used in waste treatment, as well as the catalysis of difficult chemical transformations (Akbar et al. 2013). Manganese peroxidase (EC 1.11.1.13) is manganese dependent enzyme that oxidizes organic substrates, such as phenols and phenyl radicals (Durán 2010). Laccase (EC 1.10.3.2) can oxidize different compounds, such as phenolic dyes, phenols, chlorophenols, diphenylmethanes, benzopyrenes, organophosphorus and other compounds with similar molecular structures to lignin (Shraddha et al. 2011).

Endo-1,4-β-d-glucanase (cellulase) (EC 3.2.1.4) promotes the hydrolysis of β-1,4 bonds in the amorphous regions of cellulose molecules, decreasing the degree of polymerization, exposing the microfibrils to other enzymatic attacks. Currently, fungal cellulases are used at industrial processes, emphasizing the hydrolysis of lignocellulosic biomass (Wang et al. 2012; Zhao et al. 2012). Endo-1,4-β-xylanase (xylanase) (EC 3.2.1.8) hydrolyses β-1,4 bonds of xylan substrate, also promoting the decreasing of polymerization degree (Aro et al. 2005). Filamentous fungi present high levels of xylanase when compared to yeasts and bacteria (Polizeli et al. 2011).

In another context, biosurfactants and bioemulsifiers can be employed for bioremediation of areas contaminated by oil, especially petroleum. These compounds are amphiphilic molecules with dual affinity (polar–apolar), which can be microbially produced (Pacwa-Plociniczak et al. 2011; Soberón-Chávez and Maier 2011). They are secreted either extracellular or attached to cell parts, predominantly during growth on water-immiscible substrates. This happens because the biosurfactants can reduce the surface tension at the boundary phase on water-immiscible substrates, making the substrate more readily available for uptake and microbial metabolism (Desai and Banat 1997).

Microorganisms that produce these bioactive secondary metabolites can have greater ability in the digestibility of vegetal biomass. According to microbial versatility and different possibilities to investigate this topic, the present study aimed to show some technological potential of endophytic fungi to produce not only enzymes, but also some exopolymers with biosurfactant and/or bioemulsifier activities.

## Materials and methods

### Endophytic strains

All the endophytic fungi isolates evaluated in this study were deposited at Culture Collection the Laboratory of Microbiology and Biomolecules, from the Department of Morphology and Pathology, at Federal University of São Carlos—UFSCar. The isolate 47(4), characterized as *Trichoderma camerunense* was deposited at Brazilian Culture Collection of Microorganisms from Environment and Industry (CBMAI/UNICAMP): CBMAI 2095. These endophytes were isolated from Cananeia (25°05′02′S, 47°57′42′W) mangrove forest, located at São Paulo, Brazil. Sebastianes (2010) and Sebastianes et al. (2013) described and characterized these fungi, previously. Cananeia mangrove forest is a natural reserve covering an area of 15,100 ha. Brazilian government named this site as natural reserve on July 3, 1962. This reserve contains mangroves and several other coastal ecosystems, including Atlantic Rainforest and Restinga, as well an inland forest (Sebastianes et al. 2013; Dias et al. 2010).

### Activities of ligninases

Preliminarily, ligninolytic enzyme activities were performed in a qualitative approach. Thus, all fungal isolates were cultured in BKG agar (glucose 10.0 g L^−1^; peptone 2.0 g L^−1^; yeast extract 1.0 g L^−1^; agar 20.0 g L^−1^ and guaiacol 4 mM). This screening is based on the microbial oxidation of guaiacol (Sigma Aldrich^®^) by ligninolytic enzymes after 4 to 7 days incubation at 28 °C, checking the color change of the medium from yellow to brown (D’Souza-Ticlo et al. 2006).

Positive hits in these qualitative analyses were conducted to a second round of investigation, using spectrophotometric assays aiming to quantify Lignin Peroxidase (LiP; EC 1.11.1.14), Manganese Peroxidase (MnP; EC 1.11.1.13) and Laccase (Lac; EC 1.10.3.2), after 7 days of incubation in a rotating shaker at 28 °C in ME (Malt Extract Oxoid^®^) broth 3%, in triplicates.

LiP activity was quantified using the adapted methodology from Arora and Gill (2001). It was analyzed the oxidation of veratryl alcohol (Sigma Aldrich^®^) to veratryl aldehyde, in the presence of H_2_O_2_. For the analysis of MnP, it was adopted a modified method of Kuwahara et al. (1984). MnP was determined by measuring the oxidation rate of phenol red substrate, in the presence of H_2_O_2_. Lac analyses were performed according to method described by De Pinto and Ros Barceló (1996), based on the oxidation of guaiacol.

All spectrophotometric measurements were performed using a microplate reader (Biotek Synergy HT, USA). Negative control was composed by the culture medium without the microbial inoculum. One unit of enzyme activity (U) was defined as the amount of enzyme needed to generate one (1) µmol of product reaction per minute.

### Stablishment of optimal enzymatic conditions

Aiming to stablish optimal conditions for ligninolytic activities, analyzes at different temperatures and pH ranges were performed, using an adaptation of the method developed by Heuts et al. (2007). Such analyzes were just conducted to selected strain, which could showed some prominent ligninolytic activities, at the preliminarily described assays. The reactions were conducted at the following temperatures: 37.0 and 45.0 °C, at pH 2.0 to 9.0.

### Cellulase and xylanase activities

Endo-cellulase (endo-1,4-β-d-glucanase; EC 3.2.1.4) and endo-xylanase (endo-1,4-beta-xylanase; EC 3.2.1.8) activities were monitored with commercial kits AZO-CM-Cellulose and AZO-XYLAN-BIRCHWOOD (Megazyme^®^ International, Bray, Ireland), respectively. For both analyzes, the 30 isolated were inoculated for 4 days in ME supplemented by 10.0 g L^−1^ cellulose (Celuflok 100^®^) for the cellulase activity, or 10.0 g L^−1^ xylan (Sigma^®^ X4252) for the xilanase, in triplicate.

Absorbance measurements were developed using a spectrophotometer UV/Vis (BioTek Synergy HT, USA). Negative control was composed by the culture medium, non-inoculated by the fungal strains. Calculation of activities and positive control (*Trichoderma reesei*—RUT C30) were performed according to the manufacturer’s specifications.

### Biosurfactant and bioemulsifier production assays

For screening of biosurfactant-producing isolates, all fungi were analyzed by qualitative drop-collapse technique, described by Boudour and Miller-Maier (1998). The fungal isolates were incubated, in triplicate, at ME, during 96 h. Then, the supernatant aliquots were analyzed about the presence or absence of biosurfactant activities, when spoted in the center of a thin coat of automotive engine oil disposed at polystyrene lid of a 96-microwell plate, with diameter of 8 mm. If the drop remained beaded, it was considered a negative hit. However, if the drop was collapsed, it was get a positive hit. Non-inoculated culture medium was used as negative control, while Tween 80 (Synth^®^) 2% solution was applied as positive control.

### Emulsification Indexes (E_24_)

The positive hits selected at drop-collapse assay were evaluated about their emulsification abilities, using three different apolar compounds (soy oil, automotive engine oil and hexane 85%). The isolates were cultured at ME medium during 4 days, in triplicate. Besides that, the supernatants were examined about bioemulsifiers production, according Cooper and Goldenberg (1987) methodology. The E_24_ indexes were calculated according to Fleck et al. (2000), and described as percentage values. The culture medium without inoculum was also adopted as negative control, while solution of Tween 80 (10%) was used as positive control.

### Tensiometric analysis

Positive hits obtained through drop collapse technique, were also evaluated about their tensiometric abilities, using the ring method, according to methodology described by Rodrigues et al. (2006). Therefore, the selected isolates were inoculated in the mineral medium supplemented with saccharose (5.0 g L^−1^), soy oil (5.0 mL L^−1^), peptone (2.5 g L^−1^) and yeast extract (2.5 g L^−1^), during 4 days, in triplicate. The analysis were based in the measurement of surface tension of microbial supernatants (mN/m) using a Krüss K6 tensiometer. The negative control was uninoculated culture medium.

## Results

### Ligninolytic activities

**Fig. 1:**
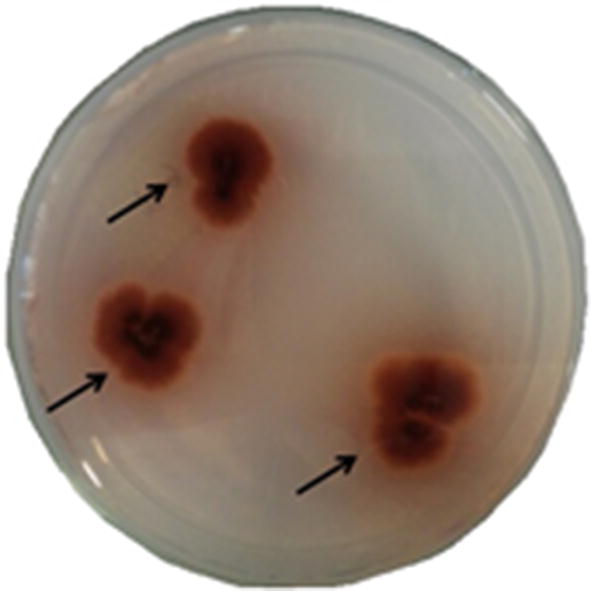
Illustrative image of a positive hit cultured in guaiacol agar (BKG). The arrows show the color change of the medium (yellow to brown), which indicates the oxidation of guaiacol by the action of the ligninolytic enzymes produced by the isolated. It is the result of 16.1—*Fusarium* sp.

Using qualitative screening applying Guaiacol Agar (BKG) medium, nineteen (19) fungal isolates showed some ligninolytic activities. As illustrated through Fig. 1, the obtained positive hits exhibited brown color under and/or around their colonies, confirming the microbial guaiacol oxidation reaction. Then, after these qualitative screening, the positive hits were analyzed about the specific activities of lignin peroxidase (LiP), manganese peroxidase (MnP) and laccase (Lac) using spectrophotometric assays.

**Fig. 2:**
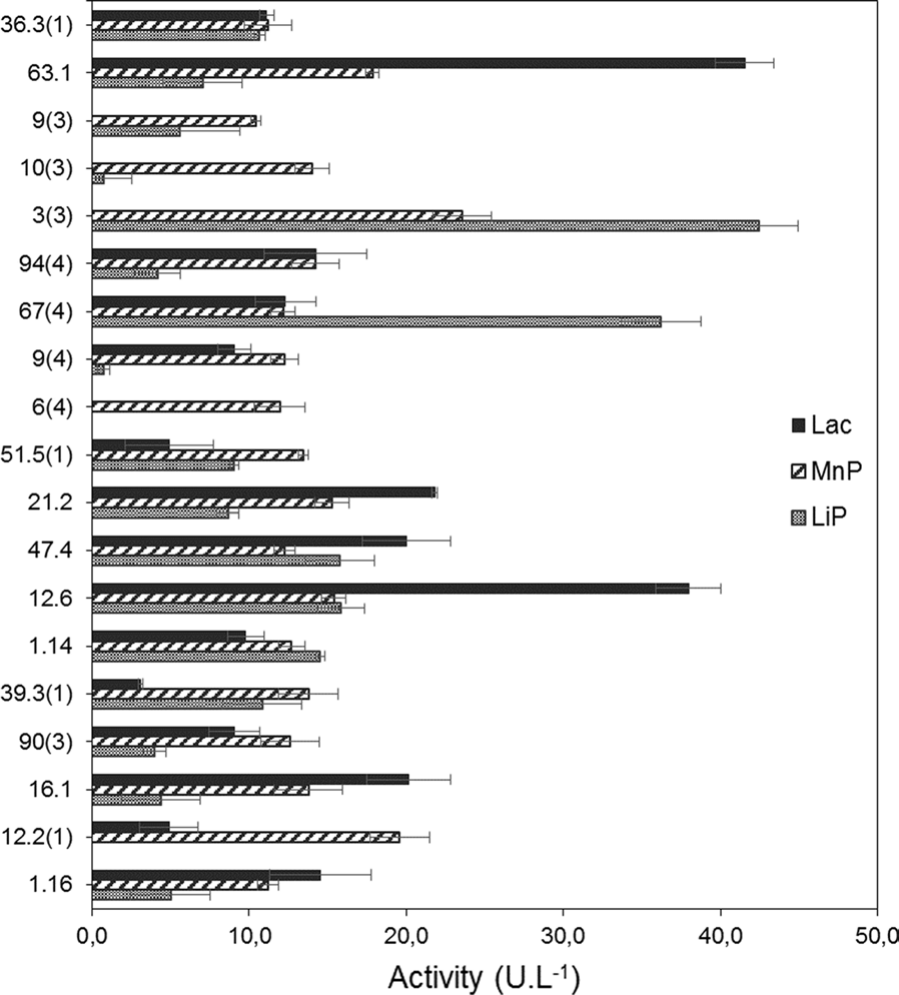
Ligninolytic activity of lignin peroxidase (LiP; EC 1.11.1.14), manganese peroxidase (MnP; EC 1.11.1.13) and laccase (Lac; EC 1.10.3.2) assays, values in U L^−1^. Results about the fungi selected positive in qualitative screening with guaiacol used like the substrate. Lac assays of isolates 9(4) and 3(3) unrealized. The data represent the means and standard errors of three measurements

LiP analysis, revealed satisfactory results for fungi 3(3)—*Fusarium sambucinum* and 67(4)—*Diaporthe* sp., showing 42.4 U L^−1^ and 36.2 U L^−1^, respectively. Three (3) isolated (12.6, 47.4 and 1.14) showed LiP activities ranging from 15.9 to 14.5 U L^−1^. All of the 19 evaluated fungi could showed some MnP activities. In this context, the isolates 3(3)—*F. sambucinum* and 12.2(1)—*Diaporthe* sp. reached the highest activity values, 23.6 and 19.6 U L^−1^, respectively. In addition, about Lac activities, the isolates 63.1—*Fusarium* sp. and 12.6—*Hypocrea lixii* showed the highest performances, 41.5 U L^−1^ and 38.0 U L^−1^, respectively (Fig. 2).

### Stablishment of optimal enzymatic conditions

**Fig. 3:**
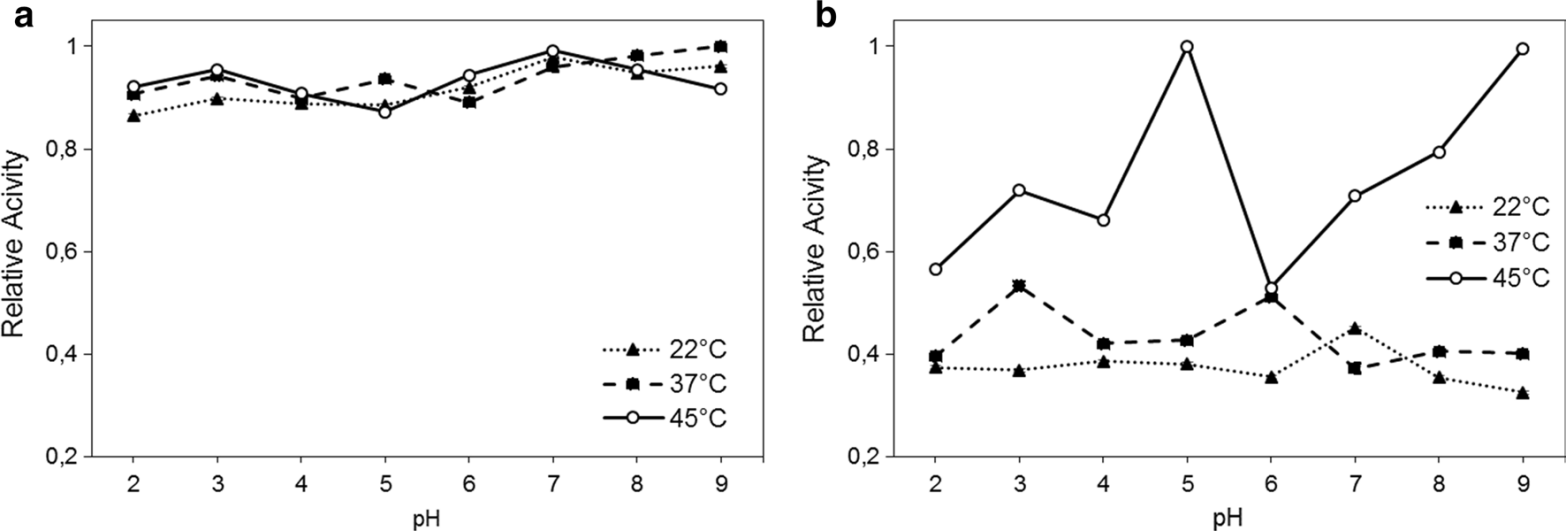
Relative activity of strain 3(3)—*Fusarium sambucinum* about its ligninolytic activities in pH range to 2.0–9.0 and three (3) temperatures (22, 37 and 45 °C) for stablish optimal conditions. **a** Lignin peroxidase (LiP) using veratryl alcohol like substrate and **b** manganese peroxidase (MnP) with phenol red was used as substrate these analyses. The data represent the means and standard errors of three measurements

It was selected the isolated 3(3)—*Fusarium sambucinum* as a model microorganism to optimize reaction conditions, in order to improve its ligninolytic abilities. This isolated was adopted as it showed prominent LiP and MnP activities, in the previous described analysis. In this context, a pH range between 2.0 and 9.0, under three (3) temperatures (22, 37 and 45 °C) were evaluated. MnP activities showed some variated values, if compared to LiP activities. It was obtained pH 9.0 at 37 °C, as the optimum conditions for LiP activity. For MnP enzyme, pH 5.0 at 45 °C was detected as the optimum condition (Fig. 3).

### Cellulolytic and xylanolytic activities

**Fig. 4:**
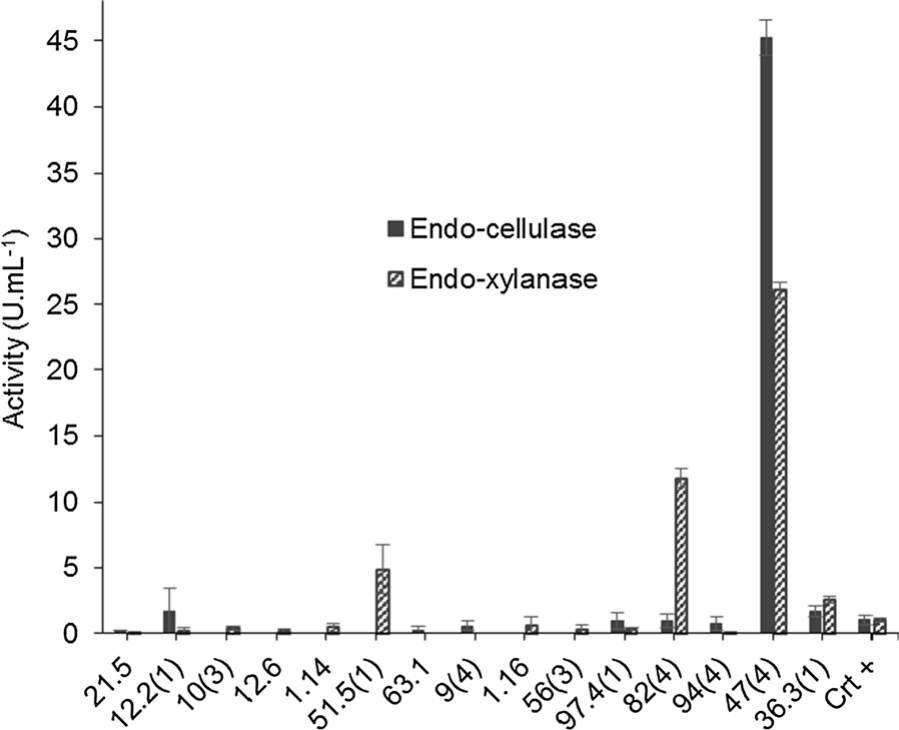
Endo-cellulases (endo-1,4-β-d-glucanase; EC 3.2.1.4) and endo-xilanases (endo-1,4-beta-xylanase; EC 3.2.1.8) activities, expressed in U mL^−1^, of positive fungi for commercial kits AZO-CM-Cellulose and AZO-XYLAN–BIRCHWOOD (Megazyme^®^ International, Bray, Ireland), respectively. Positive control performed according to the manufacturer’s specifications, using a strain of *Trichoderma reesei*—RUT C30. The data represent the means and standard errors of three measurements

All the 30 endophytic fungi were investigated about their endo-cellulase and endo-xylanase activities. Fifteen (15) fungi showed either cellulase or xylanase activities. Nine (9) isolates showed cellulolytic activities. Three (3) of them showed better performance than adopted positive control [47(4), 12.2(1) and 36.3(1)]. Thirteen (13) fungi could demonstrated some xylanolytic ability, emphasizing four (4) isolates that showed results comparable to positive control [47(4), 82(4), 51.5(1) and 36.3(1)]. The most standout cellulolytic and xylanolitic activities were showed by the isolated 47(4)—*Trichoderma camerunense*, which is affiliated with the same genera of the positive control *Trichoderma reesei* (Fig. 4).

### Biosurfactant and bioemulsifier activities

Through qualitative drop collapse screening it was possible to select fifteen (15) positive hits. Besides that, these selected fungi were analyzed about their potential to produce bioemulsifier compounds, calculating their Emulsification Indexes (E_24_) under non polar compounds. All the evaluated fungi could show some ability to emulsify automotive engine oil and soybean oil, while eight (8) isolates were able to emulsify *n*-hexane.

**Fig. 5:**
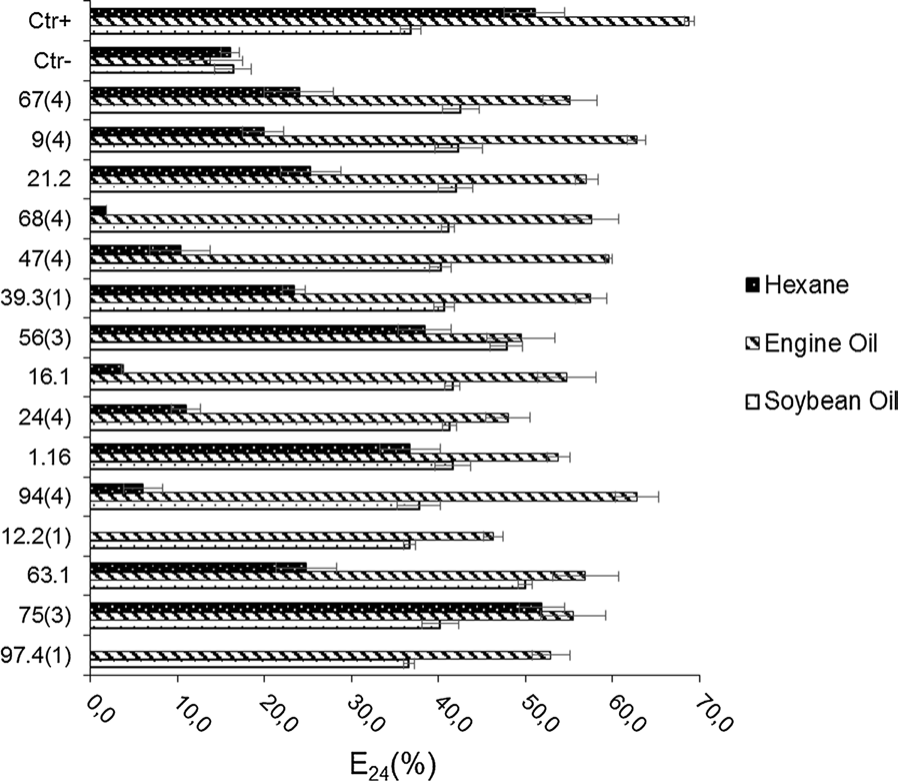
Emulsification Index (E_24_) for three apolar compounds (soybean oil, automotive engine oil and hexane 85%) for the fifteen (15) strains selected in the initial qualitative screening to the detection of potential production of metabolites with biosurfactant and bioemulsifier abilities and its controls, negative (Ctr−) and positive (Ctr+). The data represent the means and standard errors of three measurements

When automotive engine oil was used to evaluate the emulsifier potential of the fungi, it was possible to verify the most prominent E_24_ values, up to 62.8%, by the isolates 94(4)—*Diaporthe* sp. and 9(4)—*Aspergillus awamori*. Against commercial soybean oil, two isolates showed the most expressive emulsifier activities, above de positive control: 63.1—*Fusarium* sp. (50.0%) and 56(3)—*Aspergillus niger* (47.8%). For n-hexane, the best results were obtained by 75(3)—*Fusarium chlamydosporum* (51.9%) and 56(3)—*Aspergillus niger* (38.4%), above de positive control. Figure 5 illustrates the obtained E_24_ indexes under all the investigated compounds.

In this study, it was possible to select fifteen fungi about their emulsifying ability. All of them were also evaluated by tensiometric analyzes. Exception for the isolate 56(3)—*Aspergillus niger*, all of them showed some surfactant abilities. It is worth noting about the isolated 63.1—*Fusarium* sp. and 39.3(1)—*Xylaria enteroleuca*, which achieved the most prominent surface tension, 36.0 mN/m, followed by 47(4)—*Trichoderma camerunense*, 38 mN/m.

## Discussion

Industrial and environmental applications of ligninolytic enzymes are diverse and for this reason a number of studies about these enzymes are found. Although it is worth noting the studies with fungi of the genus *Fusarium* sp., *Diaporthe* sp., *Hypocrea* sp. (Tooley and Roberts 2016; Gajera et al. 2015; Lozovaya et al. 2006), which showed higher ligninolytic activities in this study. Bonugli-Santos et al. (2012) analyzed ligninolytic enzymes in three marine-derived basidiomycetes, and showed the highest LiP activity (2.234 U L^−1^) for *Tinctoporellus* sp., as well highest MnP activity (4.514 U L^−1^) for *Marasmiellus* sp. Silva et al. (2014) observed 117.33 U L^−1^ for MnP activity by *Trametes villosa*. About Lac activity, Stoilova et al. (2010) described, approximately, 1.7 U L^−1^ also for *Trametes* genus.

In the stablishment of optimal enzymatic conditions for LiP and MnP enzymes, it was observed the same patterns, which means the presence of more than one point of high activity. It was probable due the analyses were performed using the microbial supernatants, which may consist of a mixture of metabolites that influence the enzymatic activities, including the presence isoforms of enzymes that differ in the amino acid sequence and may present different optimal pH ranges and temperatures (Fernández-Fueyo et al. 2014). Moreover, the assays showed that optimal enzymatic conditions for manganese peroxidase were performed in similar pH, while the increase in temperature resulted in an improvement in its activity. However, for lignin peroxidase, changes of pH and temperature increased its activity.

Evaluating cellulolytic and xylanolytic activities, Gouvêa (2013) showed values between 1.0 and 1.5 U mL^−1^ for cellulase, as well 7.5 to 10.0 U mL^−1^ for xylanase by an *Aspergillus niger* strain. In a similar study evaluating another *A. niger* strain, it was obtained values up to 18 U mL^−1^ and 216 U mL^−1^ for cellulase and xylanase activities, respectively (Bansal et al. 2011). Michelin et al. (2010) reported xylan-degrading activities between 1.5 to 11.0 U mL^−1^ and 2.0 to 10.95 U mL^−1^, for *Aspergillus terricola* and *A. ochraceus*, respectively. Gottschalk et al. (2013) investigated xylanolytic activity in a mutant strain of *A. awamori* reached 12.9 U mL^−1^ as the maximal xylanase activity, using yeast extract as nitrogen source.

Therefore, comparing the obtained results with the literature, it was possible to select the strain 47(4)—*Trichoderma camerunense* as a potential cellulase (45.23 U mL^−1^) and xylanase (26.09 U mL^−1^) producer. According to the literature, there are microorganisms that can produce both enzymes, as described by Das et al. (2013), whose showed cellulolytic and xylanolytic activities for *Aspergillus fumigatus*. It is worth to mention that the main efforts into improve the production of second generation ethanol are focused in the cellulolytic and xylanolytic activities under lignocellulosic substrates (Mabee and Saddler 2010). Furthermore, there are many studies about cellulose activity with different species of *Trichoderma* sp. (Iqbal et al. 2011; Kirk et al. 2002). Iqbal et al. (2011) related a cellulase activity of 398 U mL^−1^ of *Trichoderma viride*.

Among the fifteen (15) fungi that showed ability to produce bioactive secondary metabolites (biosurfactant and bioemulsifier), twelve (12) also showed some enzymatic activity, corroborating about microorganisms that produce these compounds can have greater ability in the digestibility of the vegetal biomass. When comparing the obtained results among the isolated genera, it was possible to verify that *Aspergillus* sp. could be selected as a potential emulsifier producer, under the evaluated conditions of E_24_ indexes. Some species of *Candida* sp., *Aspergillus* sp., *Cladosporium* sp., *Fusarium* sp., *Ustilago* sp. and *Trichosporon* sp. were reported as able to produce compounds with these properties (Bhardwaj et al. 2013; Qazi et al. 2013; Castiglioni et al. 2009; Mulligan 2005; Desai and Banat 1997). According Lira (2014) fungal strains were able to detoxify the environment due to their abilities to join in organic matter. Kiran et al. (2009) showed biosurfactant production in *A. ustus* isolated from the marine sponge (E_24_ of 42.8%).

Endophytic fungi have systems that can breakdown complex compounds, degrading chemical pollutants and exhibiting biosorption of heavy metals (Zhang et al. 2012; Li et al. 2012; Russell et al. 2011; Xiao et al. 2010). However, the knowledge about the bioemulsifier production by endophytic fungi is still very scarce. Only few studies were reported about this ability. Lima et al. (2016) showed emulsifier index of diesel oil (52%) from *Phoma* sp. isolated from macrophytes at the Negro River, Manaus, Brazil. Other study with endophytic fungi isolated from *Myrcia guianensis* showed E_24_ values up to 75.75% (Da Silva et al. 2014).

About the results of tensiometric analysis, in a similar study, reported by Reis et al. (2004), one isolated of *Bacillus subtilis* was able to produce surface tension of 28.7 mN/m. Similarly, Qazi et al. (2013) exhibited surface tension of 32 mN/m from *Fusarium* sp. Moreover, by comparison surface tension breakdown obtained in the present study with synthetic surfactants, such SDS—sodium dodecyl sulphate, that can reduce the surface tension from 72 to 37 mN/m, it was verified that the obtained biosurfactant were efficient. This fact demonstrated that biosurfactants as potential as commercial surfactants, besides that it reduce the cost of production, has low toxicity, high biodegradability and environmental control (Kim et al. 2000).

Briefly, these results were possible to emphasize about the versatility of endophytic fungi, since enzymatic abilities, like oxidases and hydrolases that can to improve the lignocellulosic bioethanol production. Moreover, the production of metabolites with bioremediation potential, as biosurfactants and bioemulsifiers. Among the isolates, it is worth noting about the strain 47(4)—*Trichoderma camerunense*, which showed not only emulsifier and tensiometric activities, as well as a prominent cellulolytic and xylanolytic activities, turning it as a potential candidate for more investigations about future biotechnological applications.

## Data Availability

The datasets used and/or analyzed during the current study are available from the corresponding author on reasonable request.
